# An App for Detecting Bullying of Nurses Using Convolutional Neural Networks and Web-Based Computerized Adaptive Testing: Development and Usability Study

**DOI:** 10.2196/16747

**Published:** 2020-05-20

**Authors:** Shu-Ching Ma, Willy Chou, Tsair-Wei Chien, Julie Chi Chow, Yu-Tsen Yeh, Po-Hsin Chou, Huan-Fang Lee

**Affiliations:** 1 Department of Nursing Chi Mei Medical Center Tainan Taiwan; 2 College of Humanities and Social Science Southern Taiwan University of Science and Technology Tainan Taiwan; 3 Department of Physical Medicine and Rehabilitation Chi Mei Medical Center Tainan Taiwan; 4 Department of Physical Medicine and Rehabilitation Chung Shan Medical University Taichun Taiwan; 5 Department of Medical Research Chi Mei Medical Center Tainan Taiwan; 6 Department of Pediatrics Chi-Mei Medical Center Tainan Taiwan; 7 Department of Pediatrics Taipei Medical University Chi Mei Medical Groups Taipei Taiwan; 8 Medical School St George’s, University of London London United Kingdom; 9 Department of Orthopedics and Traumatology Taipei Veterans General Hospital Taipei Taiwan; 10 School of Medicine National Yang-Ming University Taipei Taiwan; 11 Department of Nursing College of Medicine National Cheng Kung University Tainan Taiwan

**Keywords:** nurse bullying, NAQ-R assessment, receiver operating characteristic curve, convolutional neural network, computerized adaptive testing

## Abstract

**Background:**

Workplace bullying has been measured in many studies to investigate its effects on mental health issues. However, none have used web-based computerized adaptive testing (CAT) with bully classifications and convolutional neural networks (CNN) for reporting the extent of individual bullying in the workplace.

**Objective:**

This study aims to build a model using CNN to develop an app for automatic detection and classification of nurse bullying-levels, incorporated with online Rasch computerized adaptive testing, to help assess nurse bullying at an earlier stage.

**Methods:**

We recruited 960 nurses working in a Taiwan Ch-Mei hospital group to fill out the 22-item Negative Acts Questionnaire-Revised (NAQ-R) in August 2012. The k-mean and the CNN were used as unsupervised and supervised learnings, respectively, for: (1) dividing nurses into three classes (n=918, 29, and 13 with suspicious mild, moderate, and severe extent of being bullied, respectively); and (2) building a bully prediction model to estimate 69 different parameters. Finally, data were separated into training and testing sets in a proportion of 70:30, where the former was used to predict the latter. We calculated the sensitivity, specificity, and receiver operating characteristic curve (area under the curve [AUC]), along with the accuracy across studies for comparison. An app predicting the respondent bullying-level was developed, involving the model’s 69 estimated parameters and the online Rasch CAT module as a website assessment.

**Results:**

We observed that: (1) the 22-item model yields higher accuracy rates for three categories, with an accuracy of 94% for the total 960 cases, and accuracies of 99% (AUC 0.99; 95% CI 0.99-1.00) and 83% (AUC 0.94; 95% CI 0.82-0.99) for the lower and upper groups (cutoff points at 49 and 66 points) based on the 947 cases and 42 cases, respectively; and (2) the 700-case training set, with 95% accuracy, predicts the 260-case testing set reaching an accuracy of 97. Thus, a NAQ-R app for nurses that predicts bullying-level was successfully developed and demonstrated in this study.

**Conclusions:**

The 22-item CNN model, combined with the Rasch online CAT, is recommended for improving the accuracy of the nurse NAQ-R assessment. An app developed for helping nurses self-assess workplace bullying at an early stage is required for application in the future.

## Introduction

### Background

Over the past several decades, prevalence rates of workplace bullying have been addressed in a wide range of different studies to investigate bullying’s potential effects on mental health [1-4]. Despite all of this attention on workplace bullying, the classification of bullying levels has still not, to this date, reached a consensus in the literature.

### NAQ-R Assessment Used for Examining Workplace Bullying

The 22-item Negative Acts Questionnaire-Revised (NAQ-R) [2,4-7] is one of the most popular tools used for examining individuals who deal with workplace bullying. Using cutting points at –0.7 and 0.7 (or <30 and <60 in the summed score), this test has been proposed to assess nurses to identify their bullying grade from one of three levels (high, moderate, and low) [4]. However, the assessment accuracy for classifying individual bullying levels is challenging and requires improvement due to type I and type II errors.

### Convolutional Neural Networks

Convolutional neural networks (CNNs) have had a significant impact within the field of health informatics [8,9]. Its architecture can be described as an interleaved set of feedforward layers implementing convolutional filters followed by reduction, rectification, or pooling layers [10-12]. For each layer, the CNN creates a high-level abstract feature. The CNN, a famous deep learning method, can improve prediction accuracy up to 7.14% [12] in classification. Accordingly, the 22-item NAQ-R, combined with the CNN technique for improving the prediction accuracy of workplace bullying, is worthy of study.

### Computerized Adaptive Testing With CNN

Computerized adaptive testing (CAT) is based on item response theory (IRT) that adapts to an examinee’s ability level [4]. The computer follows an IRT-based algorithm that provides the examinee with the next item, which can be not too hard or not too easy, for answering the next question. As such, each patient needs to answer the fewest possible items, resulting in less respondent burden and even more accurate outcomes [2]. As with all forms of web-based technology development, there has not been an online NAQ-R CAT assessment combined with CNN to assess individual workplace bullying available until now. The issue of missing responses in CAT affecting the CNN computation is one of the problems that limit the development of CAT with CNN. Another limitation for CAT is the numerous parameters within CNN, which are harder to program computer routines than traditional predictive methods, such as multiple regression analysis or logistical regression, which only have a few independent variables in their prediction models.

### Online Assessment Using Smartphones is Required

As the age of digital technology approaches, advances in mobile health (mHealth) and health communication technology are rapidly increasing [13]. Till now, there has been no app for smartphones that measures nurse bullying levels in health care settings. It is not only the complexity of the CAT procedure with multimedia illustrations embedded into the web-based module, but also the difficulty of the model’s CNN parameters that need to be transformed into the probability of classification types when the individual bullied-levels are assessed by the NAQ-R CAT. A web-based CAT with CNN app could more accurately alert individuals to alleviate their mental strain before it becomes a serious bullying-victim problem.

### Study Aims

The aims of the current study are to: (1) estimate the model’s parameters on the NAQ-R responses by the nurses; and (2) design an app for smartphones based on a website-based assessment of nurse bullying levels.

## Methods

### Data source

The study sample was recruited from three hospitals (Hospital A: 1236-bed medical center; B: 265-bed local hospital; and C: 877-bed region hospital) in southern Taiwan in August 2012. No incentive for participation was offered. A total of 960 copies of the bullying questionnaire were validated, with a return rate of 96.3% [4]. This study was approved and monitored by the Research Ethics Review Board of the Chi-Mei Medical Center. Demographic data were anonymously collected: gender, work tenure in hospitals of all types, age, marital status, and education level.

### Featured Variables

Featured variables include the 22 items in the NAQ-R in which a higher response denotes a more serious bullying problem. The input layer for each case, with 36 elements as a 6×6 image, was constructed with the 22 featured variables and the sequentially repeated responses (eg, the elements from 23 to 36 are followed from the beginning till to the end). The 960 participants were then split into training and testing sets in a proportion of 70:30, where the former was used to predict the latter. The data are shown in Multimedia Appendix 1.

### Unsupervised and Supervised Learnings

Unsupervised learning indicates agnostic aggregation of unlabeled data sets yielding groups or clusters of entities with shared similarities that may be unknown to the user before the analysis step [14,15] (eg, clustering dimensionality reduction using principal component analysis or k-mean clustering). The k-mean clustering aims to partition n observations into k clusters, in which each observation belongs to the cluster with the nearest mean [16]. Two sets of two and three categories each were clustered in comparison to this study. In contrast, supervised learning employs “labeled” training data sets (defined by the previous approach of k-mean clustering) to yield a qualitative or quantitative output through the CNN algorithm [14,17].

**Figure 1:**
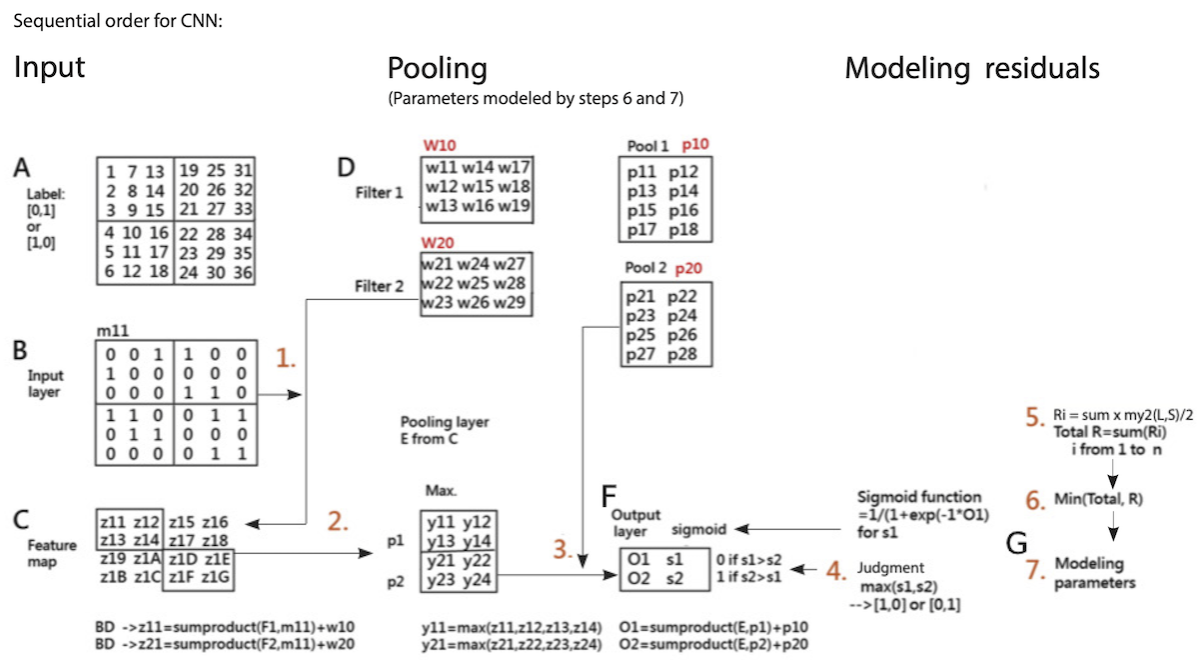
Interpretation of the CNN algorithm in Microsoft Excel. CNN: convolutional neural network.

**Figure 2:**
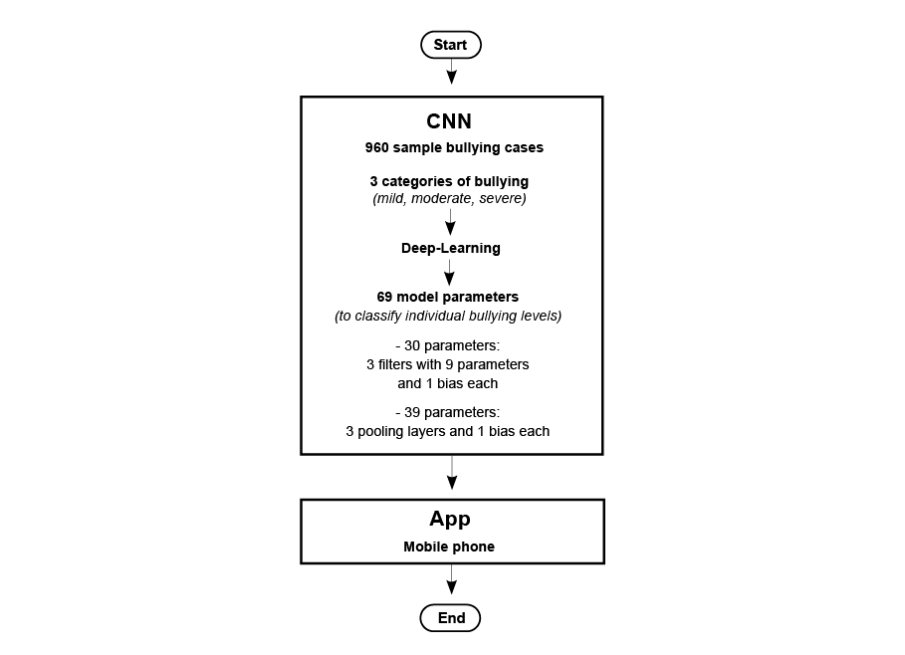
The study flowchart. CNN: convolutional neural network.

In this study, the k-mean was used as unsupervised learning for: (1) clustering participants into two classes (eg, the three categories of suspicious mild [n=918], moderate [n=29], and severe [n=13]). CNN was applied as supervised learning to build a bully prediction model for estimating the 69 parameters. See Figure 1 and 2 for more detailed information.

### CNN Applied to This Study

CNN is a variant of the standard multilayer perceptron, and it is especially used for pattern recognition compared with conventional approaches [18] due to its capability to reduce the dimension of data, extract a feature sequentially, and classify one structure of the network [19]. The basic CNN model was inspired in 1962, from the visual cortex proposed by Hubel and Wiesel [18]. To simplify the CNN concept and process, we present it in Figure 1 (see Multimedia Appendix 2 for more detailed information on interpretation).

### Tasks for Performing CNN

Task 1 is the comparison of prediction accuracies in the tree-category model. Two sets of categories (ie, 2 and 3) on 960 cases were mirrored to compare, first, the prediction accuracies (eg, the sensitivity, specificity, receiver operating characteristic (ROC) curve, and area under the curve [AUC]) using k-mean clustering. Task 2 is validation compared to the training and testing sets. We used the known responses and their corresponding labels (ie, suspicion of bullying levels) to build a model for predicting the unknown label of the specific responses. The 960 cases were split into training and testing sets in a proportion of 70:30, with the former used to predict the latter. The accuracy rates in these two sets were compared. Finally, task 3 is the app detecting bullied levels for a web-based assessment. A 22-item self-assessment app using mobile phones was designed to predict bullying levels using the CNN algorithm and the model parameters [20]. The resulting classification was based on the 22-item model.

### Statistical Tools and Data Analysis

MedCalc 9.5.0.0 for Windows (MedCalc Software, Ostend, Belgium) was used to calculate the sensitivity, specificity, and corresponding AUC using logistic regression when the observed labels and the predicted probabilities (ie, the a2 calculated by the sigmoid function in the output layer) were applied. A visual representation displaying the classification effect was plotted using the Rasch category characteristic curve (CCC) [21,22]. The study flowchart and the CNN modeling process are shown in Figure 2 and Multimedia Appendix 2, respectively.

## Results

### Demographic Data of the 960 Cases

A sample of 960 nurses was obtained for the study. The mean age of the participants was 32.7 (SD 5.8) years old, 96% (n=922) were female, and more than 57.5% (n=553) were unmarried (Table 1).

**Table 1:** Demographic data of the study sample.

Variables		n (%)
**Hospital**		
	Hospital A	542 (56.4)
	Hospital B	323 (33.6)
	Hospital C	95 (10)
**Gender**		
	Male	38 (4)
	Female	922 (96)
**Education**		
	High school	6 (0.6)
	College	464 (48.3)
	University	474 (49.3)
	Graduate school	16 (1.8)
**Marriage**		
	Unmarried	553 (57.5)
	Married	403 (42.1)
	Divorced	4 (0.4)
**Nursing grade**		
	N0	34 (3.5)
	N1	281 (29.3)
	N2	316 (32.9)
	N3	243 (25.3)
	N4	86 (8.9)
**Title**		
	Nurse	772 (80.3)
	Chief	158 (17.7)
	Leader	8 (0.8)
	Others	12 (1.2)

### Task 1: Comparison of Prediction Accuracies in the Tree-Category Model

**Figure 3:**
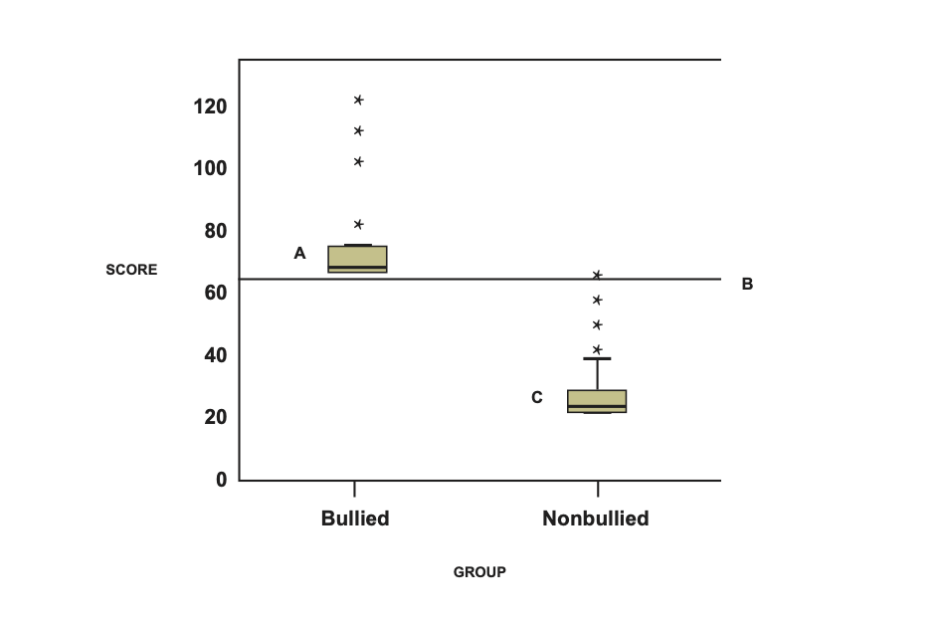
Two groups divided by the k-mean clustering. A) n=35; B) Cuttoff point set at 66 points; C) n=925.

Two groups divided by the k-mean clustering are shown in Figure 3. The cuttoff point is set at 66 points. Another visual representation displaying the classification effect is plotted using a box plot (see Figure 3).

**Figure 4:**
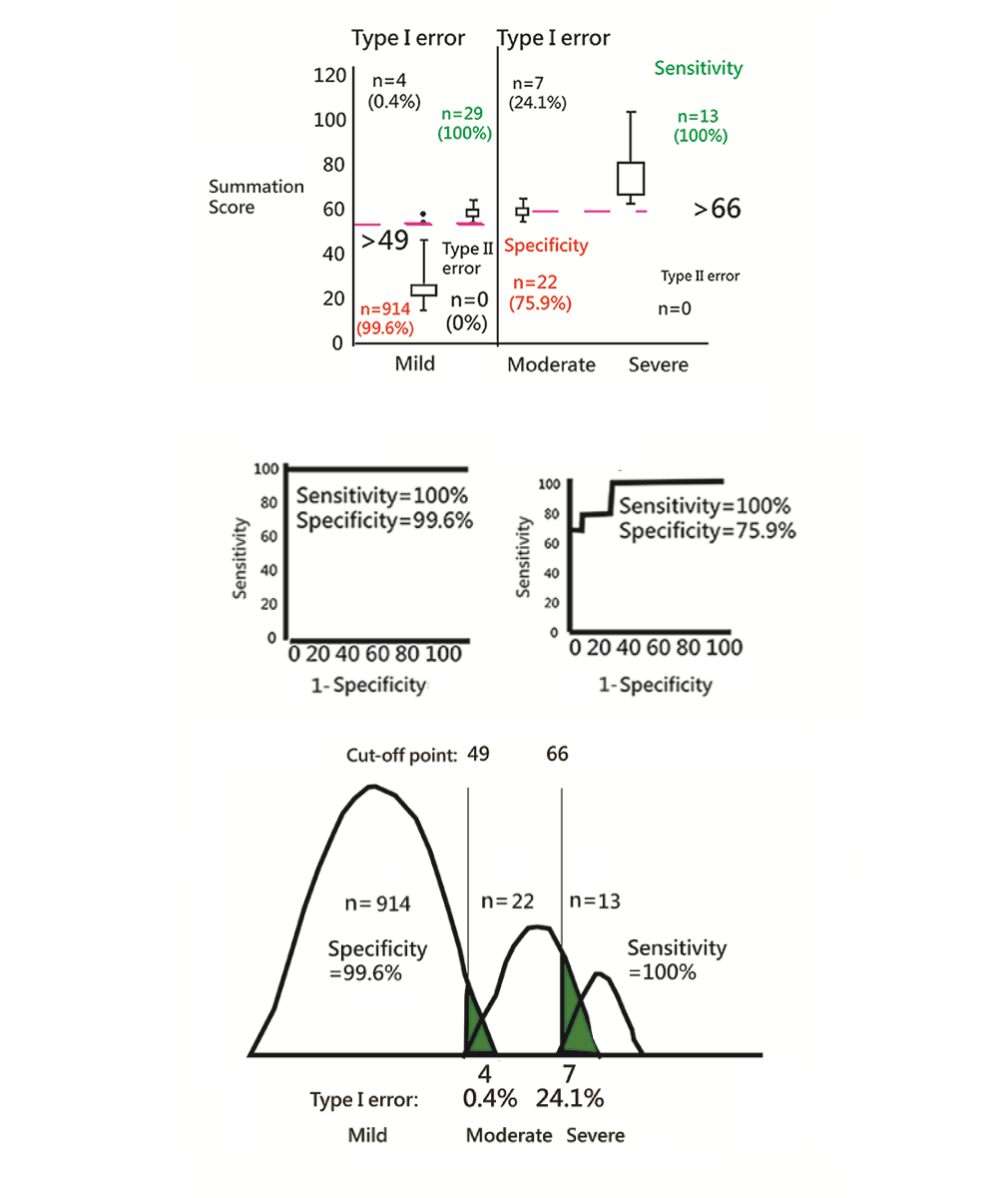
The bullied classes clustered with 3 categories using cut-off points to identify the sensitivity and specificity with AUC (area under the curve).

We can see that the 22-item model yields higher accuracy rates for three categories, with an accuracy of 94% for the total 960 cases, and accuracies of 99% (AUC 0.99; 95% CI 0.99-1.00) and 83% (AUC 0.94; 95% CI 0.82-0.99) for the lower and upper groups (cutoff points at 49 and 66) based on the 947 cases and 42 cases, respectively (see Figure 4 and Tables 2 and 3).

**Table 2:** Mild and moderate scenario applied to CNN for the prediction of nurse bullying levels.

Scenario A (22 items), ACC^a^=0.99, (n=947)	True condition			
	Positive	Negative	PPV^b^/FOR^c^	FDR^d^/NPV^e^
Positive	29	4	0.88	0.12
Negative	0	914	0	1
Sensitivity	1	—^f^	—	—
FPR^g^	0.01	—	—	—
FNR^h^ (Miss rate)	0	—	—	—
Specificity	0.99	—	—	—
AUROC^i^ (95% CI)	0.99 (0.99-1)	—	—	—

^a^ACC: accuracy

^b^PPV: positive predictive value.

^c^FOR: 1-PPV.

^d^FDR: 1-NPV.

^e^NPV: negative predictive value.

^f^Not applicable.

^g^FPR: false positive rate.

^h^FNR: false negative rate.

^i^AUROC: area under the receiver operating characteristic curve.

**Table 3:** Moderate and severe scenario applied to CNN for the prediction of nurse bullying levels.

Scenario B (22 items), ACC^a^=0.83, (n=42)	True condition				
	Positive	Negative	PPV^b^/FOR^c^	FDR^d^/NPV^e^	
Positive	13	7	0.65	0.35	
Negative	0	22	0	1	
Sensitivity	1	—^f^	—	—	
FPR^g^	0.24	—	—	—	
FNR^h^ (Miss rate)	0	—	—		—
Specificity	0.76	—	—		—
AUROC^i^ (95% CI)	0.94 (0.82-0.99)	—	—		—

^a^ACC: accuracy

^b^PPV: positive predictive value.

^c^FOR: 1-PPV.

^d^FDR: 1-NPV.

^e^NPV: negative predictive value.

^f^Not applicable.

^g^FPR: false positive rate.

^h^FNR: false negative rate.

^i^AUROC: area under the receiver operating characteristic curve.

### Task 2: Validation Compared to the Training and Testing Sets

The 700-case training set with an accuracy of 95% predicts the 260-case testing set reaching an accuracy of 97%. Interested readers are encouraged to see the study process in Multimedia Appendix 2, using the parameters modeled by the 700-case training set to predict the accuracy in the 260-case testing set.

### Task 3: App Detecting the Bullied Levels on a Web-Based Assessment

**Figure 5:**
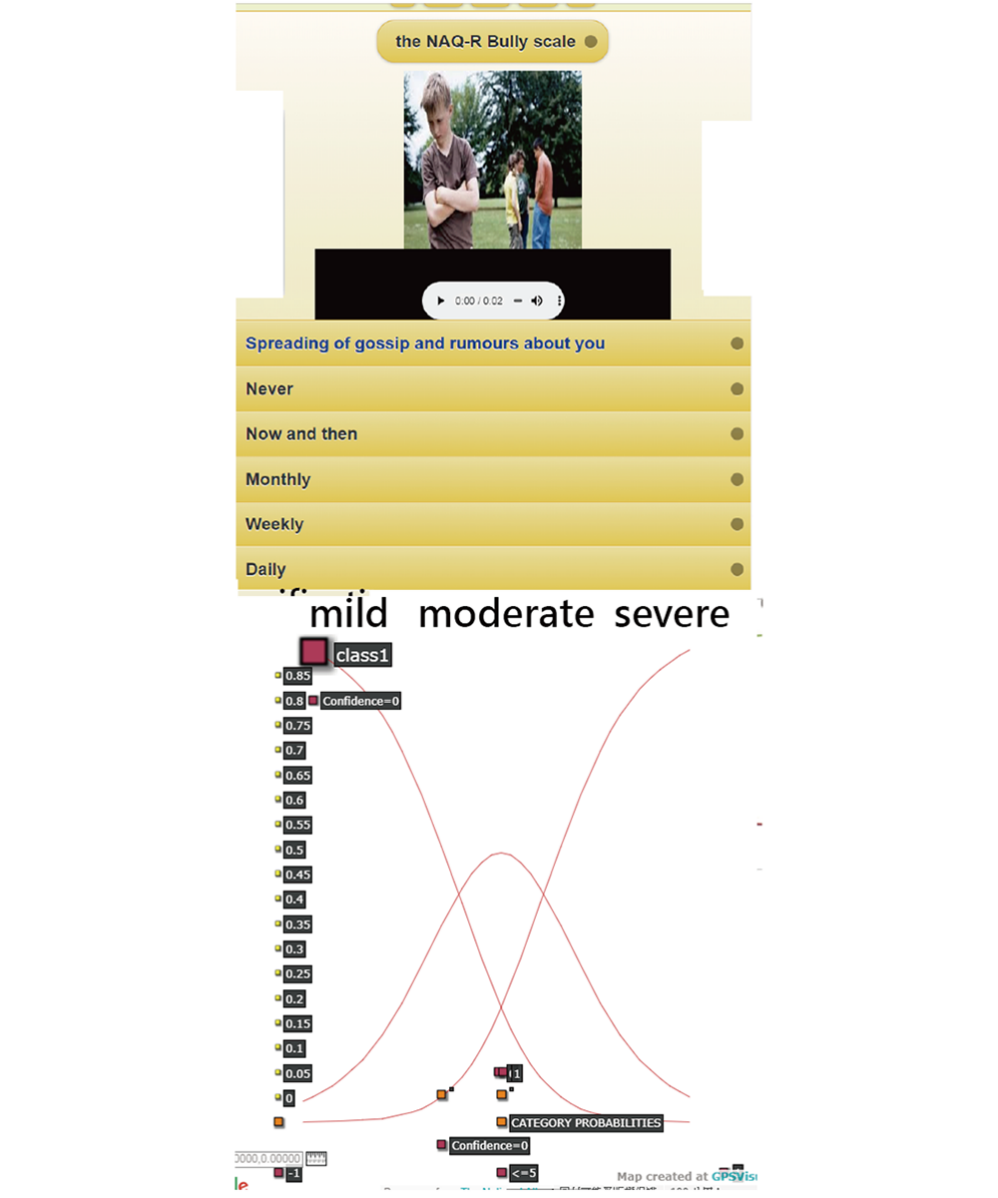
Snapshots on a mobile phone responding to questions (top) and the result (bottom) for assessing individual bullied levels.

A NAQ-R app for nurses predicting individual bullying levels was developed and demonstrated in Figure 5. One resulting example of the mild level is present at the bottom in Figure 5 on the CCC (ie, category 0 from the left-top to the right-bottom corner, category 1 in the middle, and category 2 from the left-bottom to the top-right side) based on the Rasch rating scale model [21,22], which is novel when using a visual display shown on Google Maps.

Interested readers are invited to scan the QR code to practice the NAQ-R app on their own. It is worth noting that all 69 model parameters for classifying individual bullying levels are involved in the Rasch online CAT module.

## Discussion

### Principal Findings

We observed that: (1) the 22-item model for three categories yields higher accuracy rates; and (2) the 700-case training set with an accuracy of 95% predicts the 260-case testing set reaching an accuracy of 97%. We also developed and demonstrated an NAQ-R app for nurses that predicts bullying level in this study.

The difference between the traditional score calculation method and the new model using CNN can be described as: the traditional score calculation lacks the bullying classification. As such, cutoff points are the way to classify the extent of bullying at the workplace. Nonetheless, the cutoff points drive type I (ie, false negative) and type II (ie, false positive) errors higher than the CNN algorithm.

The app created to examine and the extent of workplace bullying for individuals has two parts: (1) the Rasch CAT; and (2) classification using CNN. However, not all items answered in the Rasch CAT results were missing responses on CNN. We thus applied the Rasch rating scale model [21,22] for generating the expected responses and overcame the drawback of not having all the items answered in the CAT.

### What This Knowledge Adds to What We Already Know

The NAQ-R app has been the most widely used tool for measuring workplace bullying in the world [2,4-7]. Over 32 articles were found by searching the keywords “NAQ-R” as of September 30, 2019. However, none provided an acceptable scheme to classify the individual bullying levels (ie, mild, moderate, and severe). The previous study [4] provided a cutoff point scheme (ie, −0.7 and 0.7 logits using CAT to measure the extent of bullying) and claimed the prevalence rate of bullying for nurses was 1.5%. In this study, the cutoff points for three categories (ie, mild, moderate, and severe) are set at >49 and >66 when the total score is 110 using k-mean clustering, which are different from those set at <30 and <60 in the summed score [4] on the assumption of an equal sample size across the levels (ie, mild, moderate, and severe).

However, no matter which cutoff point scheme is applied (eg, Figure 4), misclassifications must exist due to their Type I (α) and II (1-β) errors [23]. In contrast, the CNN model can minimize Type I and II errors and improve the prediction accuracy (up to 7.14 %) [12], which is one of the features of this study.

### What it Implies and What Should be Changed

Not all questions were answered in CAT. Different from those using the mean value [9] over the entire dataset to fill the missing values, we applied the expected value in the model for each unanswered response to fill the missing data, as done in previous studies [24,25]. By doing so, the expected responses and the CNN parameters can thus be applied to classify the groups of individual bullying levels. So far, we have not seen anyone using the CNN approach to predict nurse bullying levels in the literature, which is a breakthrough, and the second feature of this study.

Over 708 articles were found using the keyword “convolutional neural network” (Title) when searching in PubMed Central as of September 23, 2019. None of the studies found used Excel (Microsoft Corp, Redmond, Washington, United States) to perform the CNN. The interpretations of the CNN concept and the process, and the parameter estimations, are shown in Figure 1 and Multimedia Appendices 2-4, which is the third feature of this study.

Furthermore, at the end of 2019, 200 papers were collected from the US National Library of Medicine National Institutes of Health when searching the keywords “computer adaptive testing.” None that were published used an online assessment with CNN suited for smartphones, and thus were not applicable for this study. We believe that more papers in the future will be published on the usefulness of online CAT with CNN, because all forms of web-based technology are rapidly increasing [13], so a need for classification assessment in clinical settings will also increase.

### Strengths of This Study

It is easy to set up an online CAT assessment form if the designer uploads relevant audio and visual files to the corresponding questions in the database. We applied the CNN algorithm along with the model’s parameters to design the routine on an app that is used to detect individual bullying levels for nurses in hospitals (see Figure 5), which is the fifth feature of this study. We have not seen any such NAQ-R [2,4-7] CAT combined with CNN implemented on mobile phones before.

As with all forms of web-based technology, advances in mHealth and health communication technology are rapidly emerging [13]. Mobile online CAT assessment is promising and worth considering in many fields of health assessment. An online CAT assessment, such as the one we developed, can be used to inform examinees quickly about when and whether they should take actions or follow-up with a psychiatrist, and how to improve their behaviors and attitudes given that their lifestyle is not changed. Mobile online CAT assessment is promising, and is worth using it to promote nurses’ health literacy. It is recommended that interested readers scan the QR codes on Figure 5, one for the app and another for the MP4, and see: (1) the details about responding to questions; or (2) the real experience of answering the 22-item NAQ-R CAT with the CNN algorithm for a website assessment.

### Limitations and Future Studies

Our study has some limitations. First, although the psychometric properties of the 22-item NAQ-R have been validated for measuring workplace bullying [2,4-7], there is no evidence to support that the 22-item NAQ-R is suitable for use on CAT assessment. We recommend additional studies using their own k-mean algorithm and CNN model to estimate the parameters and see whether a difference exists. Second, although the three classes were determined by k-mean clustering with the CNN algorithm, which can increase accuracy rates (see Tables 2 and 3), we cannot guarantee that this CNN is the only thing improving classification accuracy. Future studies are encouraged to look for other types of prediction methods that can also improve the power of the model prediction, such as Logistic regression, Naïve Bayes, Decision trees, Random Forests, and Gradient tree boosting [26-35]. They could also use other artificial neural networks, such as a Feedforward Neural Network, a Radial Basis Function Neural Network, a Multilayer Perceptron, a Recurrent Neural Network, a Modular Neural Network, or a Sequence-To-Sequence Model [36]. Third, the study was based on publications [2,4] that used the 22-item NAQ-R CAT module. All the model parameters (ie, item difficulties and step-threshold difficulties) were derived from those studies. If any environment or condition is changed (eg, for other professionals or workplaces), the result (eg, the model’s parameters) will be different from the current study and worth verifying in the future. Fourth, the NAQ-R is a one-dimensional construct. The item difficulties used to estimate the person measure were calibrated by using the Rasch Winsteps software (Winsteps.com, Chicago, United States). A person’s ability (θ) should be further estimated by the computer adaptive testing method [2,4]. Similarly, a person's ability (θ) should be known when the respondent completes the NAQ-R CAT on an app. Otherwise, the remaining items that were not answered in the CAT could not be computed for the website assessment that is used to obtain the expected responses and classify the bullying levels using the CNN algorithm. Future studies should be cautious about this matter. Fifth, the way to access the app via scanning the QR code in Figure 5. the professionally practical app should be further developed for android and IOS in the future. Finally, the study sample was taken from a nurse survey. The model parameters estimated for the NAQ-R are suitable for professionals and the workplace, but generalizing these workplace bullying assessment findings (eg, the cutoff points; see Figure 4) might be somewhat limited because the sample consisted only of nurses working at hospitals. Additional studies are needed to reexamine whether the psychometric properties of the workplace bullying assessment are like those of other worksites in/out of a hospital.

### Conclusion

The contributions in this study include: (1) overcoming the problem of missing responses that affects CNN computation and limits CAT development combined with the CNN; (2) introducing CNN availability in Microsoft Excel; (3) demonstrating an app that incorporates Rasch CAT with numerous parameters in CNN. The 22-item NAQ-R CAT is recommended for combining the parameters estimated in CNN to improve the accuracy of determining individual bullying levels. An app developed for helping nurses’ self-assess workplace bullying is at an early stage but is required for application in the future.

## Appendix

Multimedia Appendix 1 Study dataset (MS Excel).

Multimedia Appendix 2 CNN using MS Excel to interpret on Figure 1.

Multimedia Appendix 3 CNN performed in Excel.

Multimedia Appendix 4 App Online assessing nurse workplace bullying.

## Abbreviations

AUC: area under the curve
CAT: computerized adaptive testing
CCC: category characteristic curve
CNN: convolutional neural network
IRT: item response theory
mHealth: mobile health
NAQ-R: the 22-item Negative Acts Questionnaire-Revised
ROC: receiver operating characteristic
