# Supplementary material for: An App for Detecting Bullying of Nurses Using Convolutional Neural Networks and Web-Based Computerized Adaptive Testing: Development and Usability Study
Source: JMIR Mhealth Uhealth. 2020 May 20;8(5):e16747. doi: 10.2196/16747 (PMC7270851; doi:10.2196/16747)
Supplement: Multimedia Appendix 2 [file mhealth_v8i5e16747_app2.docx]

The CNN process in MS Excel:


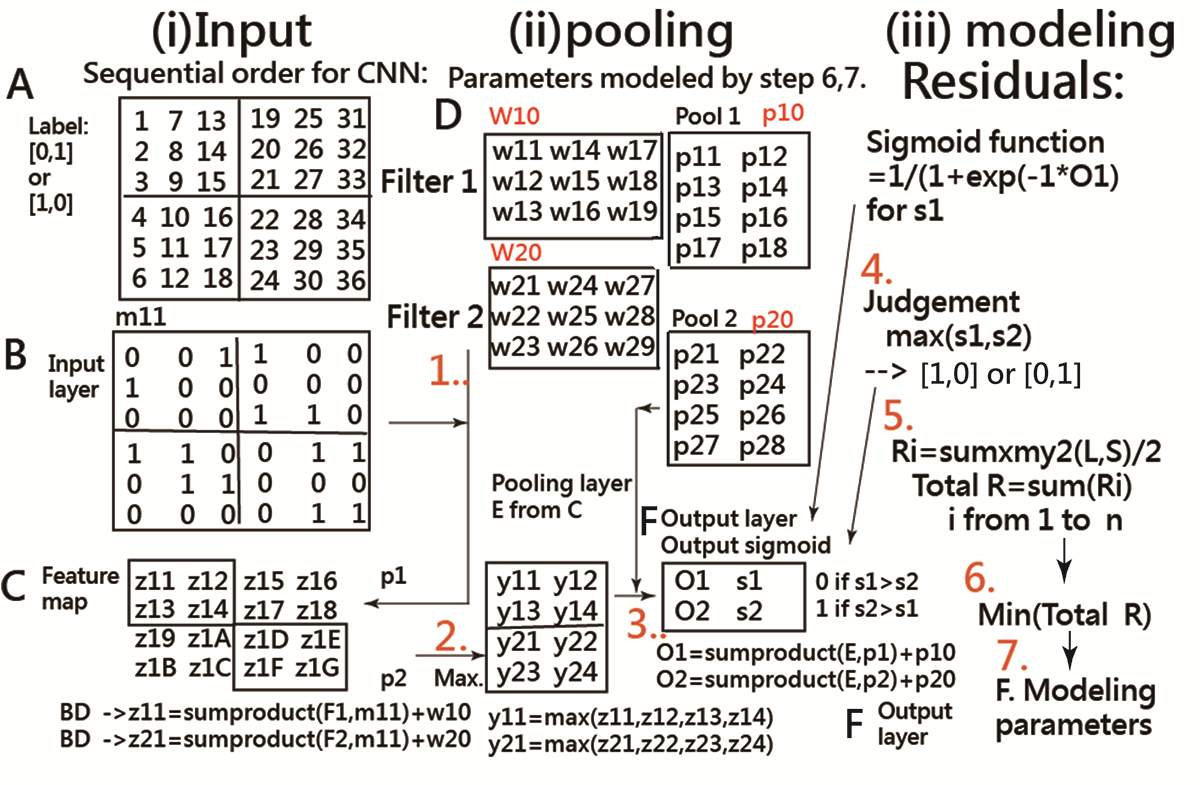


Referring to Multimedia Appendix 1, the Sequential order for CNN means the order of feature variables put in the whole metrics(as one picture) in Figure 2. I explain it with details below.

1. Referring to Multimedia Appendix 1 and Figure 1 below, 16 values from the SUMPRODUCT(W10, Input_layer) were obtained through the 16-set CNN snapshot process(i.e., {1,2,3,7,8,9,13,14,15}, {7,8,9,13,14,15,19,20,21},{13,14,15,19,20,21,25,26,27}, {19,20,21,25,26,27,31,32,33}, {2,3,4,8,9,10,14,15,16}, {8,9,10,14,15,16,20,21,22},{14,15,16,20,21,22,26,27,28},{20,21,22,26,27,28,32,33,34},{3,4,5,9,10,11,15,16,17},{9,10,11,15,16,17,21,22,23},{15,16,17,21,22,23,27,28,29},{21,22,23,27,28,29,33,35,35},{4,5,6,10,11,12,16,17,18},{10,11,12,16,17,18,22,23,24},{16,17,18,22,23,24,28,29,30},{22,23,24,28,29,30,34,35,36} by observing the sequential order at the top 36 cells).
2. Similarly, other 16 values from the SUMPRODUCT(W20, Input_ layer) were obtained.
3. A total 32 values were produced via the two SUMPRODUCT () formulas at the bottom using panels B and D as the step 1, resulting in 2-set feature map C from W10 and W20, resepectively.
4. Two-set(p1 and p2) 32 values in (3) can be divided into eight elements in pooling layer(E) by extracting four each in feature map(C) from those 32 values(p1 and p2) sequentially using the maximum function via step 2 and the bottom formulas y11 and y21.
5. Finally, the output layer at the left-bottom corner can be yielded by the 2-set pooping parameters(p10 and p20) and the eight elements in the polling layer(E) using the two functions of SUMPRODUCT(p10, E) and SUMPRODUCT(p20, E),see the O1 and O2 in output layer.
6. Judgement was made by the steps below:
7. O1 and O2 in output layer were transformed into the probability(s1 and s2) in a range from 0 to 1 through the sigmoid function, see the right-top panel.
8. Select the larger one between s1 and s2 as the classification(e.g., s1>s2 as predicting label 1, and vice versa, see step 4.
9. Modeling the parameters in the CNN model is present in steps from 5 to 7.
