# Supplementary material for: An App for Detecting Bullying of Nurses Using Convolutional Neural Networks and Web-Based Computerized Adaptive Testing: Development and Usability Study
Source: JMIR Mhealth Uhealth. 2020 May 20;8(5):e16747. doi: 10.2196/16747 (PMC7270851; doi:10.2196/16747)
Supplement: Multimedia Appendix 3 [file mhealth_v8i5e16747_app3.docx]

**Multimedia appendix 3**

CNN performed in Excel at https://youtu.be/wLRI2lwQQJc
