# Supplementary material for: An App for Detecting Bullying of Nurses Using Convolutional Neural Networks and Web-Based Computerized Adaptive Testing: Development and Usability Study
Source: JMIR Mhealth Uhealth. 2020 May 20;8(5):e16747. doi: 10.2196/16747 (PMC7270851; doi:10.2196/16747)
Supplement: Multimedia Appendix 4 [file mhealth_v8i5e16747_app4.docx]

**Multimedia appendix 4**

App Online assessing nurse workplace bullying at http://www.healthup.org.tw/irs/irsin_e.asp?type1=60
